# Circulating multi-omic signature of disease severity and cholangiocarcinoma in primary sclerosing cholangitis

**DOI:** 10.1097/HC9.0000000000001008

**Published:** 2026-07-15

**Authors:** Ghada Nouairia, Adam Schumacher, Annika Bergquist, Martin Cornillet

**Affiliations:** 1 Department of Medicine, Huddinge, Unit of Gastroenterology and Nutrition, Karolinska Institutet, Stockholm, Sweden; 2 Department of Upper Gastrointestinal Disease, Division of Hepatology, Karolinska University Hospital, Stockholm, Sweden; 3 Center for Infectious Medicine, Department of Medicine, Huddinge, Karolinska Institutet, Stockholm, Sweden

**Keywords:** cholangiocarcinoma, inflammatory bowel disease, machine learning, multi-omic integration, primary sclerosing cholangitis, weighted network analysis

## Abstract

**Background:**

Primary sclerosing cholangitis (PSC) is a chronic cholestatic liver disease often associated with inflammatory bowel disease (IBD) that can progress to cirrhosis or cholangiocarcinoma (CCA), both carrying a poor prognosis. The mechanisms driving disease progression remain poorly understood. We aimed to identify circulating multi-omic signatures linked to PSC severity (alkaline phosphatase, bilirubin, fibrosis), IBD, and CCA to elucidate underlying biology and discover potential biomarkers for risk stratification and disease monitoring.

**Methods:**

We quantified 737 proteins, 1083 metabolites, and 4573 miRNAs using plasma from 33 patients with PSC with various clinical presentations. We applied machine learning-driven analysis and network-based modeling for the data integration and analysis of associations. Parameters robustly identified in both methods were considered for tailored pathway enrichment analysis.

**Results:**

We identified circulating multi-omic profiles associated with PSC disease severity, IBD, and CCA. IBD-specific alterations were subtle; however, we noted associations between microbial-derived metabolites and colorectal cancer-related miRNAs. Severe PSC shared molecular features with PSC-CCA, indicating early activation of malignant pathways in PSC. Severe PSC was characterized by immune-interacting and epithelial-interacting proteins, bile acid and glutathione-related metabolites, and miRNAs regulating fibrosis, inflammation, extracellular matrix remodeling, and cell cycle control, overlapping with PSC-CCA. In established PSC-CCA, we observed coordinated alterations in oncogenic miRNAs, growth factor-related proteins, and depletion of sulfated phenolics and methylxanthines, reflecting extracellular matrix remodeling, inflammatory microenvironment reprogramming, and impaired hepatic detoxification. Overall, pathway enrichment analysis revealed an infection-like and cancer-associated signature defining PSC pathogenesis.

**Conclusions:**

Circulating multi-omic profiles capture the key features of PSC severity and associated CCA. These findings provide mechanistic insights and identify potential biomarkers for cancer risk stratification and disease monitoring using PSC.

## Graphical abstract

**Figure:**
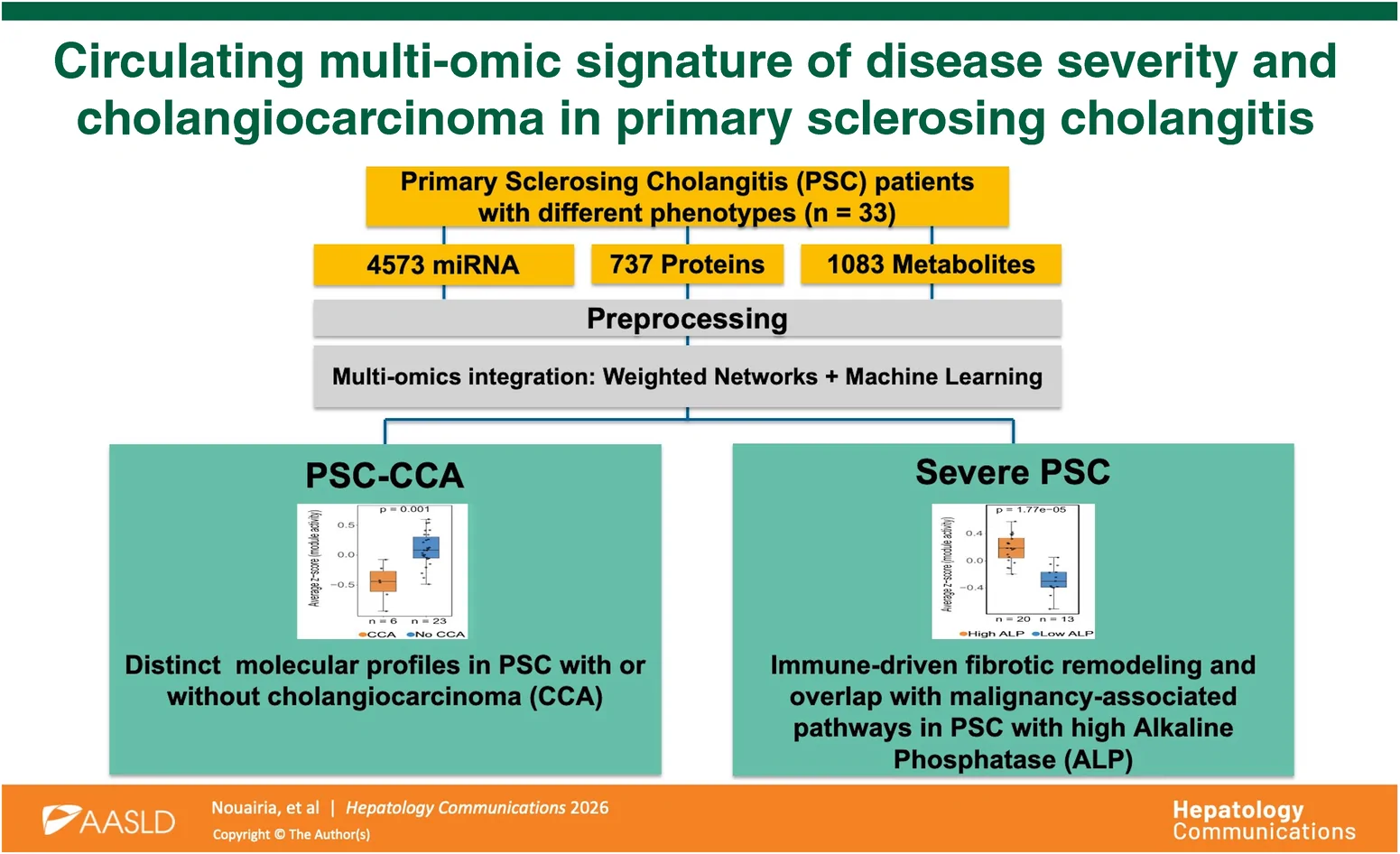

## INTRODUCTION

Primary sclerosing cholangitis (PSC) is a chronic liver disease characterized by bile tract inflammation that leads to cirrhosis and is associated with an elevated risk of biliary cancer. Its course is highly heterogeneous, ranging from indolent to rapid progression, leading to liver failure and hepatobiliary malignancy.^1^ Cancer is the leading cause of mortality in PSC, with cholangiocarcinoma (CCA) developing in 7%–20% of patients over their lifetime and reflecting a risk increase of up to 400-fold relative to the general population.^1^ CCA is frequently diagnosed at advanced or unresectable stages owing to the lack of sensitive and specific surveillance tools. Conventional tumor markers, such as carbohydrate antigen 19-9 (CA19-9) and imaging modalities, have limited accuracy in distinguishing CCA from benign inflammatory or dysplastic lesions in PSC, which complicates early detection.^1^ As a result, the prognosis remains poor, with median survival measured in months when curative resection or liver transplantation is not feasible.^1^ Approximately 70% of PSC patients also have concurrent inflammatory bowel disease (IBD), which complicates their clinical course and management of the disease. The pathophysiological links between gut and liver manifestations remain poorly understood, and the frequent coexistence of PSC and IBD raises the question of whether these represent 2 distinct diseases or a single multifaceted immune disorder.^2^

The early identification of disease trajectories is crucial for risk stratification and patient management. Despite the availability of prognostic tools such as liver stiffness measurement, imaging modalities, and scoring systems, their ability to accurately stratify individual risk and capture the full complexity of PSC remains limited.^1^^,^^3^ The disease may arise from a complex interplay of genetic, immune, and environmental factors. It represents a distinct immunobiological entity sharing features with, but differing from, classical autoimmune diseases.^1^ In addition, genetic susceptibility loci, microbiome dysbiosis, and altered bile acid metabolism contribute to the development of the disease.^4^ Although the field acknowledges both the fragmented nature of existing findings and the need for multi-omic integration, comprehensive cross-layer integration remains scarce.^5^ In diseases such as PSC, in which immune responses, bile acid dysregulation, and fibrosis interact dynamically, multi-omics could potentially elucidate these interactions and reveal biomarkers that reflect the underlying pathology more accurately than a single biological layer.

Integrative multi-omic analysis, including circulating multi-omics, has demonstrated substantial value in biomarker discovery, disease monitoring, and patient stratification across several complex diseases.^6^ By combining diverse biological layers, these approaches can provide a comprehensive view of disease mechanisms by identifying the cross-layer biomarker signatures. Advanced computational frameworks such as Weighted Gene Co-expression Network Analysis^7^ (WGCNA) allow unsupervised detection of modules representing biological networks and pathways without prior phenotypic input, thereby facilitating data-driven hypothesis generation. Importantly, WGCNA identifies co-varying molecular modules across heterogeneous data types, capturing coordinated biological mechanisms underlying complex, multi-layered diseases such as PSC. Recently, artificial intelligence (AI) and machine learning (ML) have played an increasingly critical role in the integration of multi-omic data, enabling the capture of complex biological signals beyond traditional methods. Data Integration Analysis for Biomarker discovery using latent variable approaches for omics studies (DIABLO)^8^ is a supervised multi-omic integration method designed to identify correlated features across omics layers that are strongly associated with clinical outcomes or disease trajectories. This method is effective for selecting co-varying outcome-explaining modules and excels in characterizing subtle multifactorial traits that are difficult to disentangle with classical statistical models.^9^ Moreover, ML methods are advancing cancer research^10^ and using ML for CCA diagnosis and prognosis has been identified as a priority.^3^ ML-driven multi-omic data integration holds the promise of resolving disease phenotypes and risk factors, emphasizing its value in PSC research.

To evaluate the potential of circulating multi-omics as biomarkers for risk stratification and longitudinal disease monitoring in PSC, we applied ML-driven integrative analysis along with complementary network modeling to plasma-derived multi-omic data, including miRNAs, proteins, and metabolites, from patients with PSC. We aimed to identify the molecular signatures associated with specific clinical features of PSC, including PSC severity, coexisting IBD, and the presence of CCA.

## METHODOLOGY

### Cohort description

We enrolled 33 PSC patients across multiple clinical profiles, including PSC without IBD (n=8), PSC with CCA (n=6), high alkaline phosphatase (ALP) (>2.85 µkat/L, n=20), high bilirubin (>20 μM/L, n=16), and advanced fibrosis (stages 3–4, n=19). Clinical and outcome data were prospectively collected with a follow-up duration of 24 months. Power analysis reached 70%–80% power to detect large effects (*d* ≥ 0.8) in unbalanced groups (IBD and CCA comparisons) and ~70% power for moderate effects (*d*=0.5) in balanced comparisons (ALP, bilirubin, fibrosis).

### Data analysis

Using plasma from the patients described above, we quantified 737 proteins (Olink, Sweden), 1083 metabolites (Metabolon, USA), and 4573 mature and precursor miRNAs (Bulk Sequencing, Bioinformatics and Expression Analysis Core Facility, Sweden). Global metabolomic profiling was performed by Metabolon using an untargeted ultra-high-performance liquid chromatography–tandem mass spectrometry (UHPLC-MS/MS) platform. Data processing, peak identification, and normalization were conducted using Metabolon’s standardized pipeline. The miRNA reads were annotated using miRBase v22^11^ and counted. Only experimentally validated miRNAs (based on miRTarBase,^12^ 2025) were included in the downstream analysis. Proteins and metabolites with missing measurements in ≥8% of samples were excluded, along with miRNAs with >30% invariant counts. Protein and metabolite expression values were log2-transformed for normalization. Data quality control was performed in R (v4.3.1), including quality assessment, variance structure, outlier identification, and normality testing. Sample-wise distribution plots were inspected to confirm comparable intensity distributions across samples (Supplemental S1A, B, http://links.lww.com/HC9/C404). Unsupervised methods further evaluated the data structure, batch effects, and confounders: singular value decomposition (SVD) using ChAMP^13^ (v2.36.0), hierarchical clustering, and k-means clustering (factoextra^14^ v1.0.7). No systematic batch-associated variance was detected (Supplemental S1C–E, http://links.lww.com/HC9/C404). Mann–Whitney tests were performed on individual omics layers for each PSC phenotype comparison.

### Omic integration

Multi-omic data integration was performed following 2 methods using the WGCNA^7^ (v1.71) and mixOmics^15^ (v6.22.0) packages.

To identify coordinated molecular profiles across omics layers with WGCNA, we constructed weighted co-expression networks on a concatenated dataset including miRNA, proteomic, and metabolomic features. All features were *z*-score standardized before concatenation to ensure comparability across platforms. A weighted unsigned network was constructed using pairwise Pearson correlations, with adjacency defined as |correlation|^β, such that both positive and negative correlations contributed based on their absolute magnitude. The soft-thresholding power was set to β=7 to approximate scale-free network topology according to standard WGCNA criteria. The adjacency matrix was transformed into a topological overlap matrix (TOM), and modules were identified using hierarchical clustering with dynamic tree cutting. Module eigengenes were defined as the first principal component of the features within each module. Associations between module eigengenes and clinical traits were assessed using Pearson correlation. *p*-Values were adjusted using the Benjamini–Hochberg procedure across all tested module–trait pairs. Module–trait associations were considered significant at |*r*| > 0.6 and adjusted *p* < 0.01. For trait-associated modules, hub features were defined based on module membership (kME), calculated as the correlation between each feature and its corresponding module eigengene. Hub stability was evaluated using bootstrap resampling (200 iterations), and the stability score was defined as the proportion of resamples in which |kME| > 0.7.

We used supervised multi-omics integration using DIABLO (Data Integration Analysis for Biomarker discovery using Latent cOmponents) to identify correlated latent components across omics datasets. Model parameters were optimized using repeated leave-one-out cross-validation (LOOCV). The number of components and sparsity parameters (keepX) for each omics block were tuned by minimizing balanced error rate (BER) across block-specific grids of selected features. Final phenotype-specific keepX values were chosen based on cross-validated performance. For each comparison, a small balanced hold-out subset (n=4) was set aside before model fitting and used for descriptive assessment of generalization. Final models were then fitted on the full training dataset using the optimized parameters.

### Pathway enrichment analysis

Pathway enrichment was performed by integrating the results of the ML-based (DIABLO) and network-based (WGCNA) analyses. First, all DIABLO-selected variables and all features from the WGCNA modules that were most strongly correlated with the phenotype were compiled for each omic layer. These features were then mapped to the Kyoto Encyclopedia of Genes and Genomes (KEGG)^16^ (v114.0) compatible identifiers: miRNAs were converted to validated target genes using miRTarBase, proteins were mapped through universal protein resource (UniProt) identifiers (with KEGG handling conversion to Entrez IDs), and metabolites were assigned KEGG compound IDs, acknowledging partial mapping where relevant.

For each omics, over-representation analysis (ORA) was conducted independently using KEGG pathways with Benjamini–Hochberg (BH) correction (clusterProfiler^17^ v4.14.6; org.Hs.eg.db^18^ v3.20.0). A classical KEGG-based ORA was chosen to ensure adequate metabolite coverage, which was not achievable with alternative resources.

To obtain a unified multi-omic signal, pathway-level *p* values from the 3 omic-specific analyses were combined using Stouffer’s method (as implemented in multi-GSEA^19^), followed by BH correction. Pathways with a combined FDR < 0.05 were retained, and pathways enriched across multiple omics were prioritized to avoid bias toward large feature sets (eg, miRNAs). The integrated enrichment landscape was visualized using a tree plot and clustering pathways based on the similarity of their contributing features. Within each cluster, representative drivers were defined as variables present in ≥75% of the member pathways, allowing identification of molecular entities that consistently drive the multi-omic enrichment signal. The full analysis framework is summarized in Figure 1.

**FIGURE 1:**
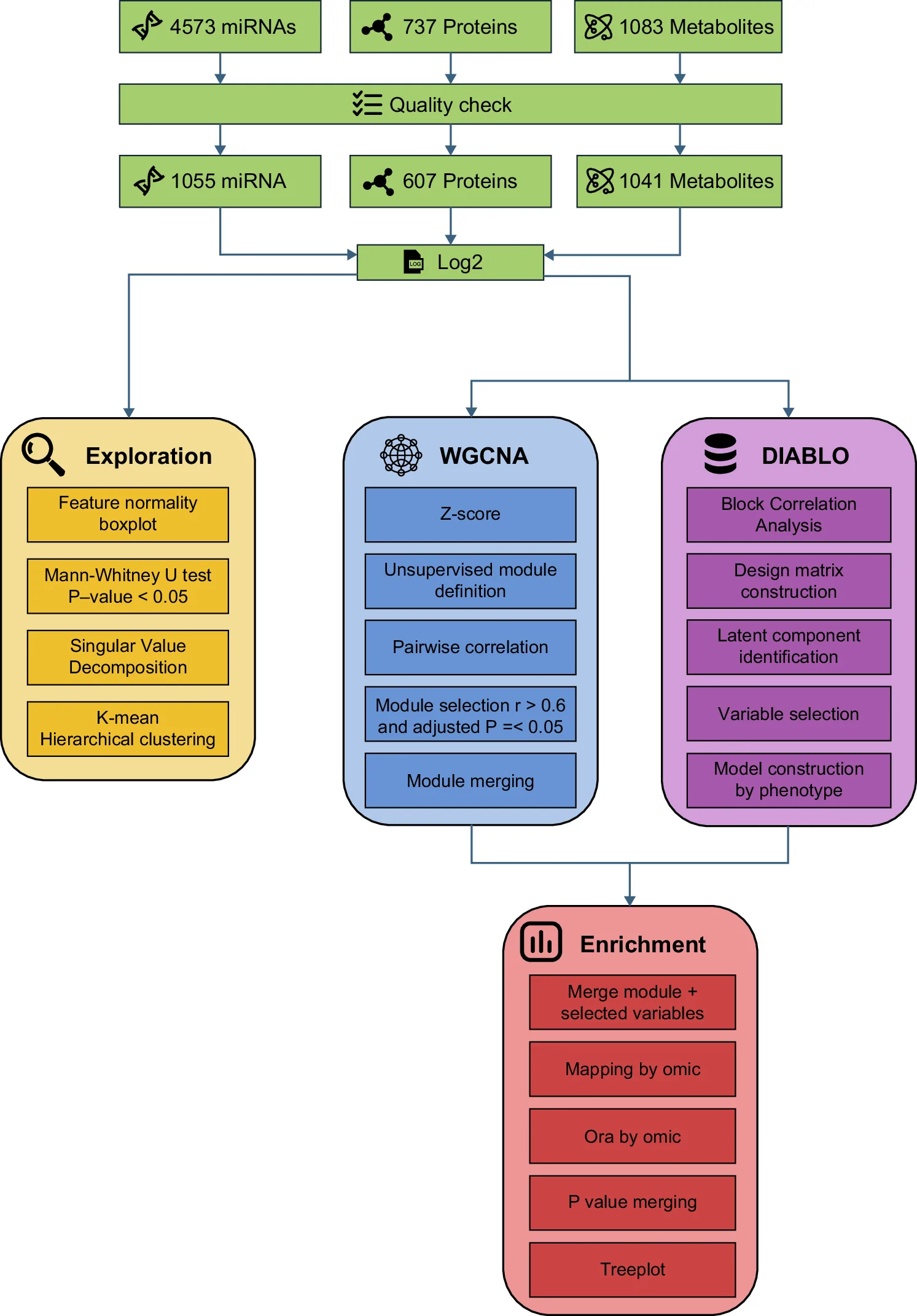
Experimental study design. Abbreviations: DIABLO, Data Integration Analysis for Biomarker discovery using latent variable approaches in omics studies; ORA, over-representation analysis; WGCNA, Weighted Gene Co-expression Network Analysis.

### Ethics, data management, and AI use

All research procedures were conducted in accordance with the Declaration of Helsinki and the Declaration of Istanbul. The study was approved by the Swedish Ethical Review Authority. Written informed consent was obtained from all participants before study inclusion.

All analysis scripts are publicly available at GitHub (https://github.com/GhadaNOUAIRIA/multiomics_integration). The datasets generated and analyzed in this study are available in the FigShare repository (DOI:10.6084/m9.figshare.30896051). Clinical and multi-omics data were handled in compliance with European Union and Swedish data protection regulations.

The authors disclose the use of artificial intelligence tools (large language models) during manuscript preparation to assist with language editing, clarity, and formatting. AI tools were not used for data collection, statistical analysis, figure or content generation, or interpretation of results. All AI-assisted content was critically reviewed and validated by the authors, who take full responsibility for the accuracy and integrity of the work.

## RESULTS

### PSC clinical phenotypes captured by circulating multi-omics

The study included 33 individuals with PSC, of whom 70% were male, with a mean age of 39.1 ± 12.3 years (Table 1). To identify omics features associated with each PSC phenotype, we sub-grouped the cohort according to the presence of IBD and CCA and levels of liver damage indicators (bilirubin, ALP, and fibrosis) (Table 2). We noticed that patients with PSC-IBD had significantly higher ALP and bilirubin levels than those without IBD (*p* < 0.01), consistent with more pronounced cholestasis. Singular value decomposition showed a large independence of single-omic datasets from major potential confounders, including age, sex, IgA and IgG levels, and steroid use (Supplemental S1D, http://links.lww.com/HC9/C404). Single-omics analyses identified miRNAs, proteins, and metabolites associated with PSC phenotypes (Supplemental S1C, http://links.lww.com/HC9/C404 and Supplemental S2A, http://links.lww.com/HC9/C405), highlighting the complementary information captured by each molecular layer, further supported by k-means and hierarchical clustering (Supplemental S1E, F, http://links.lww.com/HC9/C404). In parallel, multi-omic integration revealed strong concordance across layers (integration scores of 0.82–0.92; Figure 2A), indicating that the overall biological profiles were robust and not driven by any single data type. Next, we integrated the datasets using weighted network analysis (WGCNA; see the Methods section), which grouped patients into distinct clusters partially aligned with the clinical phenotypes (Figure 2B). For example, patients with less severe disease tended to cluster together (cluster 5), whereas patients with and without IBD or CCA were distributed across multiple clusters, suggesting heterogeneous biological profiles underlying similar clinical phenotypes. Module–trait analysis revealed strong correlations between specific multi-omic modules and disease phenotypes (Figure 2C). Hierarchical clustering of modules and PSC phenotypes showed that CCA and IBD were closely mapped in cluster iv (Figure 2D). Notably, ALP, bilirubin, and fibrosis status phenotypes were consistently and strongly associated with the 2 modules (Green and Brown) within cluster iii (*r*=0.58–0.82, *p* < 0.001; Figure 2D), highlighting shared molecular alterations linked to fibrogenesis, cholestasis, and liver injury. Although the network construction was purely data-driven and modules were defined independently of prior biological annotation, the Green module was enriched for immune receptors, chemokines, and stromal/angiogenic markers and was therefore termed the “immune–stromal module,” whereas the Brown module was dominated by bile acid and steroid hormone metabolism and was termed the “bile acid–steroid module” (Supplemental S5, http://links.lww.com/HC9/C408). Overall, our analysis defines multi-omic profiles of PSC patients by capturing key disease-related alterations.

**TABLE 1:** Clinical characteristics of individuals with PSC

Clinical parameters	
Sex: N (%)	F: 10 (30) M: 23 (70)
Age, mean (SD)	39.1 (12.3)
BMI, mean (SD)	23.3 (3)
Bilirubin, mean (SD)	38.8 (43.2) μM/L
ALP, mean (SD)	4.9 (3.8) µkat/L
ALAT, mean (SD)	2.1 (1.7) µkat/L
CRP, mean (SD)	10.8 (21.5) mg/L
Symptoms^a^: N (%)	14 (42)
Liver transplantation at follow-up: N (%)	8 (24)

^a^ Symptoms: self-reported symptoms (jaundice, pruritus, fever, or fatigue).

Abbreviations: ALAT, alanine aminotransferase; ALP, alkaline phosphatase; BMI, body mass index; F, females; M, males; N, Number; SD, standard deviation.

**TABLE 2:** Comparative clinical characteristics across PSC subgroups

Clinical parameter	Group	Group	*p*
PSC vs. PSC with CCA	PSC-CCA	PSC	
N (%)	6 (18)	27 (82)	
Sex: N (%)	M: 18 (67) F: 9 (33)	M: 5 (83) F: 1 (17)	0.7
Age, mean (SD)	41.8 (15.4)	38.5 (11.8)	0.2
BMI, mean (SD)	22 (3)	23.7 (2.9)	0.17
ALP, mean (SD)	8 (4.1)	4.2 (3.4)	0.07
Advanced fibrosis (stages 3–4) Mild fibrosis (stages 1–2), N (%)	Advanced: 3 (50) Mild: 3 (50)	Advanced: 16 (60) Mild: 11 (40)	0.9
Bilirubin, mean (SD)	66.6 (59)	32.5 (37.5)	0.22
IBD, N (%)	6 (100)	16 (59)	0.15
**PSC with IBD vs. PSC without IBD**	**PSC-IBD**	**No IBD PSC**	
N (%)	22 (67)	11 (33)	
Sex: N (%)	M: 4 (36) F: 7 (64)	M: 19 (86) F: 3 (14)	0.01
Age (SD)	39 (12.4)	40 (13)	0.7
BMI (SD)	23 (2.3)	25 (4)	0.17
ALP, mean (SD)	6 (4.1)	3 (1.7)	0.006
Advanced fibrosis (stages 3–4) Mild fibrosis (stages 1–2), N (%)	Advanced: 14 (64) Mild: 8 (36)	Advanced: 5 (45) Mild: 6 (55)	0.46
Bilirubin, mean (SD)	50.7 (48.5)	14.7 (8.5)	0.002
**Advanced fibrosis (stages 3–4) vs. mild fibrosis (stages 1–2)**	**Advanced fibrosis**	**Mild fibrosis**	
N (%)	19 (58)	14 (42)	
Sex: N (%)	M: 15 (79) F: 4 (21)	M: 8 (57) F: 6 (43)	0.3
Age (SD)	37.8 (10.6)	40.8 (14.5)	0.5
BMI (SD)	23.9 (3.2)	22.4 (2.4)	0.1
ALP (SD)	5.8 (3.4)	3.6 (4)	0.03
Bilirubin (SD)	57.3 (47.3)	13.5 (17.1)	0.001
IBD, N (%)	14 (74)	8 (57)	0.5
**High ALP (>2.85 µkat/L) vs. low ALP (<2.85 µkat/L)**	**High ALP**	**Low ALP**	
N (%)	20 (61)	13 (39)	
Sex: N (%)	M: 15 (75) F: 5 (25)	M: 8 (61.5) F: 5 (38.4)	0.6
Age (SD)	39.1 (13.2)	39 (11.3)	0.9
BMI (SD)	23 (2.3)	23.9 (4)	0.5
Advanced fibrosis (stages 3–4) Mild fibrosis (stages 1–2), N (%)	Advanced: 15 (75) Mild: 5 (25)	Advanced: 4 (31) Mild: 9 (69)	0.03
Bilirubin (SD)	56.4 (47.6)	11.5 (7.1)	0.0004
IBD, N (%)	16 (80)	6 (46)	0.1
**High bilirubin (>20 μM/L) vs. low bilirubin (<20 μM/L)**	**High bilirubin**	**Low bilirubin**	
N (%)	16 (48.5)	17 (51.5)	
Sex: N (%)	M: 12 (75) F: 4 (25)	M: 11 (65) F: 6 (35)	0.7
Age (SD)	35.75 (9.2)	42.2 (14.2)	0.1
BMI (SD)	23.8 (3.8)	22.7 (1.7)	0.3
Advanced fibrosis (stages 3–4) Mild fibrosis (stages 1–2), N (%)	Advanced: 15 (94) Mild: 1 (6)	Advanced: 4 (23) Mild: 13 (77)	0.00006
ALP (SD)	6.3 (3.3)	3.5 (3.8)	0.006
IBD: N (%)	13 (81)	9 (53)	0.1

*Note:* ALP measurements are in microkatal per liter (µkat/L), and bilirubin measurements are in micromolar per liter (µM/L). *p*-Values were calculated using Fisher's exact test.

Abbreviations: ALP, alkaline phosphatase; BMI, body mass index; CCA, cholangiocarcinoma; IBD, inflammatory bowel disease; N, number; PSC, primary sclerosing cholangitis; SD, standard deviation.

**FIGURE 2:**
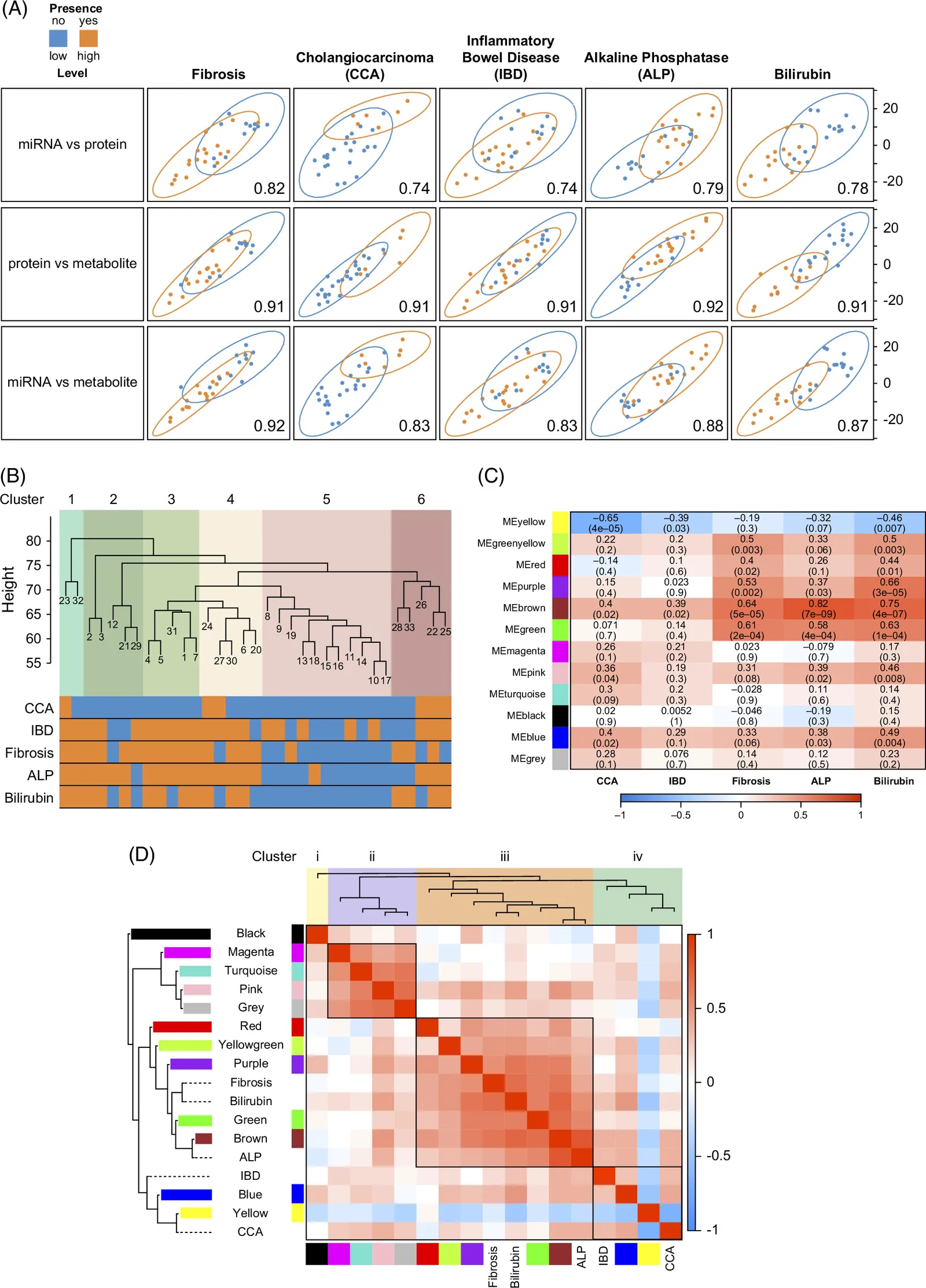
Integrated multi-omics aligns with CCA, IBD, and PSC severity. (A) Pairwise-omic correlation plots across PSC phenotypes. The figure is organized into 3 comparison layers (miRNA–proteins, proteins–metabolites, miRNA–metabolites). Individuals were plotted on DIABLO latent components with confidence ellipses, and the correlation of omic blocks was displayed. The color indicates the presence of IBD, CCA (orange = presence, blue = absence), and the level of ALP, bilirubin, or fibrosis (orange = high, blue = low). (B) Data-driven hierarchical clustering of patients (top) accompanied by a clinical phenotype heatmap (bottom). Clusters are numbered and shown in separate colors, and the heatmap color code (orange and blue) follows that of A. (C) Heatmap of WGCNA-derived modules and PSC phenotypes correlations (Pearson) with associated *p* values. Color intensity reflects the strength and direction of association. (D) Correlation matrix (Pearson) of hierarchically clustered WGCNA modules and PSC phenotypes. The dendrograms show structured module–phenotype clusters. Abbreviations: ALP, alkaline phosphatase; CCA, cholangiocarcinoma; DIABLO, Data Integration Analysis for Biomarker discovery using latent variable approaches in omics studies; IBD, inflammatory bowel disease; PSC, primary sclerosing cholangitis; WGCNA, Weighted Gene Co-expression Network Analysis.

### Molecular signature of the fibrosis–cholestasis axis severity

Next, we analyzed the multi-omic dataset, contrasting milder versus more severe PSC. We selected ALP as a representative marker of the fibrosis–cholestasis–liver damage axis, which was consistent with their module–trait clustering (Figure 2D). The results from the multi-omic integration analysis comparing bilirubin and fibrosis levels are provided in the Supplemental Information (Supplemental S3, http://links.lww.com/HC9/C406 and S4, http://links.lww.com/HC9/C407, respectively).

The bile acid–steroid (Brown) and immune–stromal (Green) modules were significantly associated with ALP (adjusted *p*=1.4e^−7^ and 0.008, respectively), showing strong correlations between their components and ALP status (*r*=0.66–0.87; *p* ≤ 1.4e^−30^, Figure 3A). A total of 510 biological entities constituted these modules, consisting mainly of proteins (n=286; 56%) and metabolites (n=176; 35%), whereas miRNAs contributed modestly (n=48; 9%) (Supplemental S5, http://links.lww.com/HC9/C408). As biological interpretation may vary across analytical strategies, we also applied the ML-based integrative method (DIABLO, see the Methods section) to link ALP status to multi-omic signatures. Integrating all omics layers showed clearer separation of patients by PSC severity, mirrored by ALP levels, outperforming similar analysis on single-omic blocks (Figures 3B, C and Supplemental S2B, http://links.lww.com/HC9/C405). The clustering was driven by 160 features, of which half were proteins (n=80), while miRNAs and metabolites contributed equally (n=40; 25%). Mir589, NECTIN2, and taurocholate were the main contributors by omics block, associated with higher ALP levels, whereas mir208b-3, SCF, and guanin were the main contributors of each block associated with lower ALP levels (Figure 3D). We also computed the DIABLO-derived cross-layer correlation matrix (Supplemental S6A, http://links.lww.com/HC9/C409) and visualized correlations ≥0.7 (Figure 3F). One cluster linked multiple conjugated/sulfated bile acids with epithelial injury and immune proteins (CXADR, KIM1, HAVCR2, TNFRSF10A, and IL-18R1), consistent with combined cholestasis and inflammatory activation. A second cluster, including glucuronate, CDCP1, CCL20, HGF, SCAMP3, miR-4322, and sphingomyelin, suggests a separate axis of epithelial remodeling and cytokine–lipid signaling.

**FIGURE 3:**
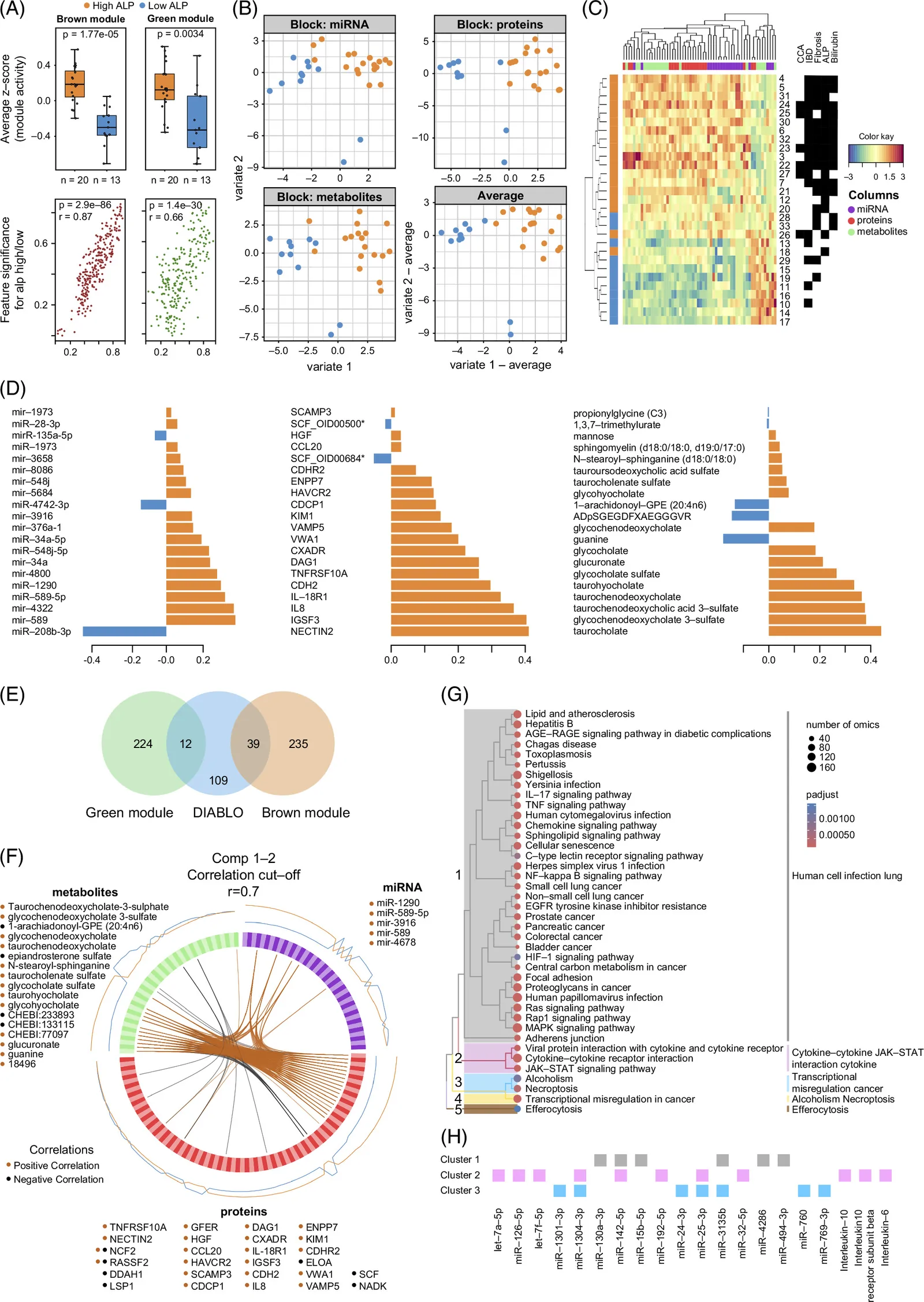
Robust multi-omic signatures characterize severity in PSC. (A) Boxplots of bile acid–steroid (Brown) and immune–stromal (Green) module activities comparing patients with severe versus mild PSC, defined by high versus low ALP levels (top plots). *p*-Value is based on Mann–Whitney tests. Correlation between module membership of each module feature and the strength of its association with ALP level for the bile acid–steroid (left) and immune–stromal (right) modules. (B) Samples plotted on the first 2 DIABLO latent components, for individual omic-blocks and averaged across layers, clearly separating high versus low ALP groups. (C) Clustered heatmap of ML-selected features (*z*-scores), annotated by ALP level (left column, color code follows A) and omics layer (top row), with PSC phenotypes heatmap on the right (black = presence or high level, white = absence or low level). (D) Key discriminant features driving the ML-based integration (first 2 latent components in DIABLO) and associated with high (orange) or low (blue) ALP levels. (E) Overlap between ALP-associated features as defined by DIABLO (blue) and WGCNA analysis (Brown and Green modules). (F) Circos plot displaying significant positive and negative correlations among key miRNAs, proteins, and metabolites associated with PSC severity. (G) Treeplot of KEGG pathways enriched across variables identified by WGCNA and DIABLO analysis. Pathways are grouped by similarity and ranked by adjusted significance and presence across all omic layers. (H) Heatmap summarizing the predominant contributors within each enriched pathway cluster. Only clusters supported by at least 2 terms are displayed. *Isoforms of proteins. Abbreviations: ALP, alkaline phosphatase; CCA, cholangiocarcinoma; DIABLO, Data Integration Analysis for Biomarker discovery using latent variable approaches in omics studies; IBD, inflammatory bowel disease; KEGG, Kyoto Encyclopedia of Genes and Genomes; ML, machine learning; PSC, primary sclerosing cholangitis; WGCNA, Weighted Gene Co-expression Network Analysis.

To summarize our findings, we compared outputs from WGCNA and DIABLO, identifying 51 shared features that consistently contributed to the biological characterization of PSC severity (Figure 3E). Based on these results, we noticed a set of immune-interacting and epithelial-interacting proteins (CCL20, IL8, TNFRSF10A, NECTIN2, MIC-A/B, FUCA1, and CKAP4) together with bile acid–related metabolites (sphingosine, sphinganine, androsterone glucuronide, and deoxycholic acid glucuronide) (Figure 3D and Supplemental S5, http://links.lww.com/HC9/C408). Decreased levels of glutathione-related metabolites indicate oxidative stress and impaired redox balance. Several fibrosis-associated and inflammation-associated miRNAs (miR-4800, miR-29a family, miR-1290, and miR-21) interfere with fibrogenesis and cholangiocyte proliferation. Glutathione-related metabolites decreased in patients with high ALP levels, consistent with enhanced oxidative stress and impaired redox balance. In addition, we identified miRNAs (eg, let-7 family and miR-126) that are known to regulate oncogenic pathways intersecting with *KRAS* and *FGFR2* mutations frequently observed in CCA.^20^^,^^21^ Given that PSC is a premalignant condition with a high risk for CCA,^3^ these miRNAs may underscore the molecular link between disease severity and malignant transformation. In addition, several proteins (IL6, IL8, CCL3, CCL20, HG, and AREG) and metabolites (glycocholate sulfate, taurocholate, and sphingomyelin) associated with high ALP levels are tightly linked to alterations in *CDKN2A*, *SMAD4*, and *HER2/ERBB2* genes, which are prevalent in CCA.^22^^,^^23^ Therefore, these multi-omic patterns linked elevated ALP in PSC not only to an active cholestatic-inflammatory state but also to malignant processes.

Finally, we developed an integrative pathway analysis framework to link the PSC severity signature to a mechanistically annotated pathway. This analysis revealed that the underlying biological perturbations reassembled infection-related mechanisms (viral and bacterial, cluster 1), the activation of JAK–STAT signaling (clusters 1 and 2), and a malignant-like transcriptional signature (clusters 1 and 3) (Figure 3G and Supplemental S7, http://links.lww.com/HC9/C410). By extracting the features contributing to at least 75% of the pathway terms within each cluster, we identified several leading miRNAs (eg, let-7 family) and cytokines (IL6 and IL10) (Figure 3H). In summary, the fibrosis–cholestasis–liver damage axis revealed a multi-omic signature with similarities to infectious-like and malignant-related processes.

### Subtle multi-omic IBD profile revealed by integrative ML

Because PSC with and without concomitant IBD have been proposed to represent distinct disease entities with differing etiologies and pathophysiologies,^2^ we next investigated whether multi-omic profiling could provide additional mechanistic insight. The subtle profile of PSC-IBD evaded detection by weighted networks (Figure 2C), but ML-based integration revealed a hidden signature (Figures 4A, B). miRNAs drove the discrimination, outperforming the integrated model, reflecting their broader coverage (full miRNA sequencing) relative to the limited protein and metabolite quantified. Immune-associated and inflammation-associated proteins, including CCL20, HGF, and VCAN, were among the largest contributors to the PSC-IBD group signals, indicating divergent regulation of immune signaling and tissue remodeling pathways (Figure 4C). In parallel, altered levels of microbial-derived metabolites, such as phenylacetate and indole propionate, were detected, suggesting differences in the gut microbiome metabolic outputs. Several miRNAs have been identified (eg, miR-1290, miR-4800) and have been previously implicated in promoting colorectal cancer cell proliferation and hepatocellular carcinoma, respectively,^24^^,^^25^ suggesting that molecular changes in PSC-IBD reflect an early carcinogenic state and provide a rationale for the heightened cancer predisposition seen in PSC-associated IBD^26^ (Figure 4C and Supplemental S8, http://links.lww.com/HC9/C411). The proteomic layer showed differential expression of IL8 and CCL20, reflecting a chemokine axis increasingly recognized as a hallmark of severe IBD and a driver of liver disease progression in PSC.^27^ These findings indicate that IBD in PSC is associated with combined immune activation and microbiota-linked metabolic remodeling, consistent with recent evidence implicating the gut–liver axis disruption in PSC pathogenesis,^26^ whereas non-IBD PSC reflects a more quiescent inflammatory state. ML integration proved effective in aggregating weak but concordant signals across omics layers, overcoming the limitations of sample size and data imbalance, and detecting subtle systemic molecular patterns underlying PSC and IBD differences.

**FIGURE 4:**
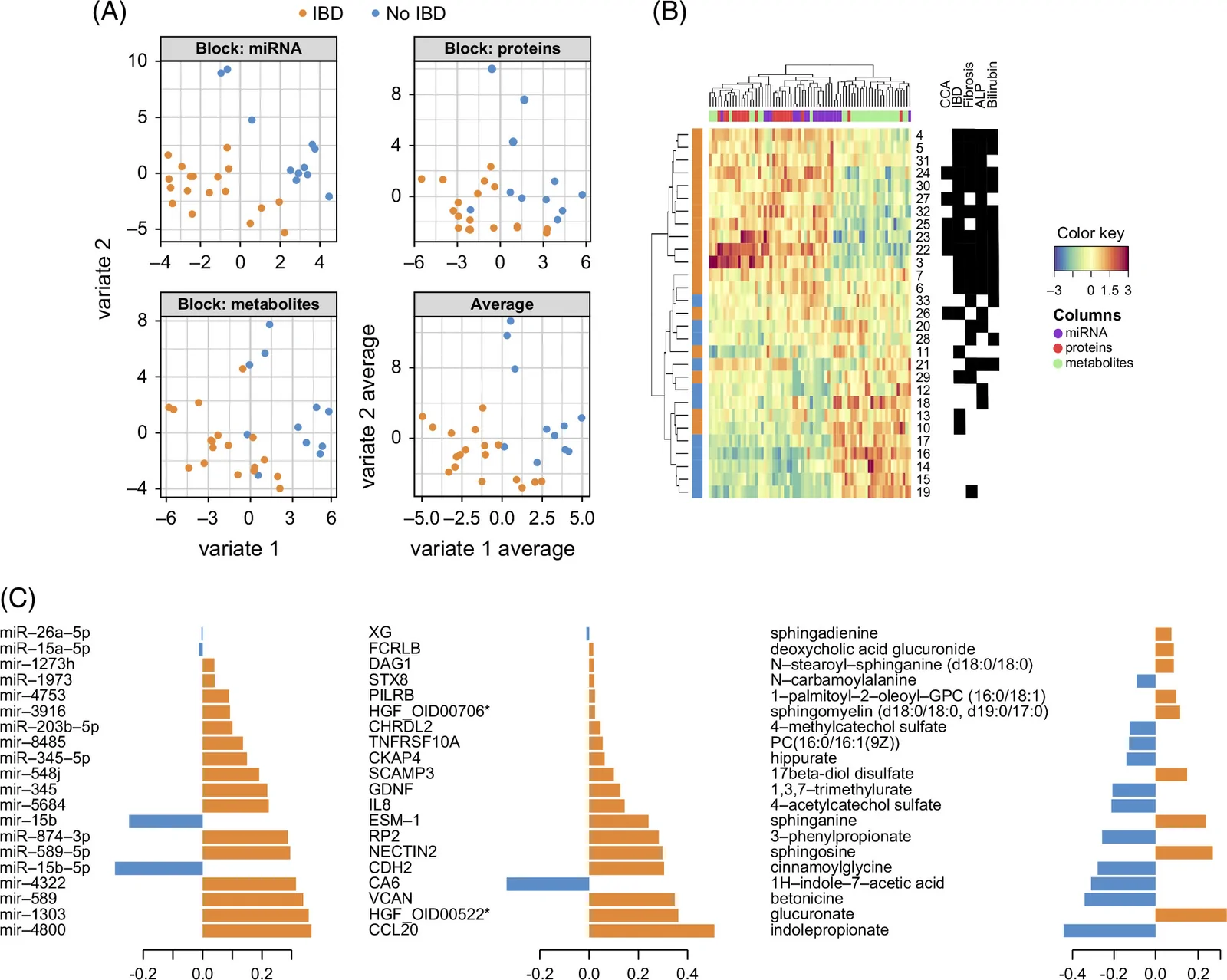
Integrative multi-omic analysis reflects weak molecular signatures of PSC-associated IBD. (A) Samples plotted on the first 2 DIABLO latent components, for individual omic-blocks and averaged across layers, separating IBD presence (orange) or absence (blue). (B) Clustered heatmap of ML-selected features (*z*-scores), annotated by IBD presence (left column, color code follows A) and omics layer (top row), with PSC phenotypes heatmap on the right (black = presence or high level, white = absence or low level). (C) Key discriminant features driving the ML-based integration (first 2 latent components in DIABLO) and associated with IBD presence (orange) or absence (blue). Abbreviations: ALP, alkaline phosphatase; CCA, cholangiocarcinoma; DIABLO, Data Integration Analysis for Biomarker discovery using latent variable approaches in omics studies; IBD, inflammatory bowel disease; ML, machine learning; PSC, primary sclerosing cholangitis.

ML-based integration demonstrates enhanced sensitivity for detecting subtle PSC phenotypes, such as concomitant IBD, relative to the clearer molecular patterns observed in other PSC phenotypes, such as PSC-CCA.

### A strong multi-omic signature of PSC-CCA

We next focused on identifying multi-omic signatures associated with CCA in patients with PSC, a clinically critical question with implications for disease monitoring and therapeutic development. Both network-based and ML-based analyses revealed a robust molecular signature of PSC-CCA. The Yellow module showed reduced expression in PSC-CCA (*r*=−0.66; adjusted *p*=1e^−4^, Figure 5A), while ML-based integration distinctively separated PSC with and without CCA (Figures 5B, C and Supplemental S2B, http://links.lww.com/HC9/C405). This module was predominantly composed of host–microbial co-metabolites, xenobiotic and amino-acid catabolites, and was therefore termed the “microbial–xenobiotic module.”

**FIGURE 5:**
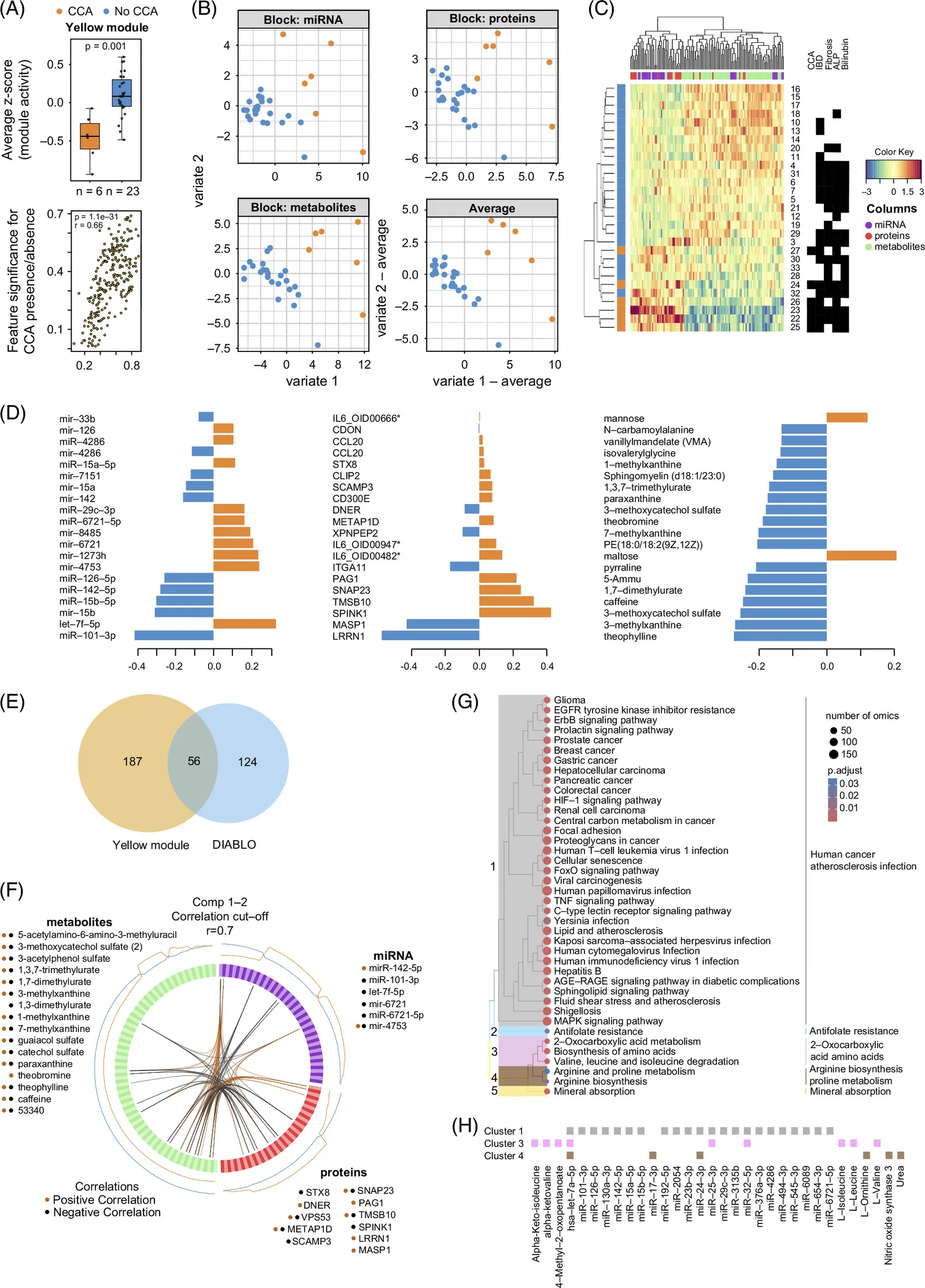
Multi-omic integration identifies molecular drivers of CCA in PSC. (A) Boxplots of the microbial–xenobiotic (Yellow) module activity comparing patients with and without CCA (top plot). *p*-Value is based on Mann–Whitney tests. Correlation between module membership of each module member and the strength of its association with CCA presence (bottom plot). (B) Samples plotted on the first 2 DIABLO latent components, for individual omic-blocks and averaged across layers, clearly separating PSC-CCA from PSC without CCA groups. (C) Clustered heatmap of ML-selected features (*z*-scores), annotated by CCA presence (left column) and omics layer (top row, color code follows A), with PSC phenotypes heatmap on the right (black = presence or high level, white = absence or low level). (D) Key discriminant features driving the ML-based integration (first 2 latent components in DIABLO) and associated with the presence (orange) or absence (blue) of CCA. (E) Overlap between features associated with CCA as defined by DIABLO (blue) and WGCNA analysis (Yellow module). (F) Circos plot displaying significant positive and negative correlations among key miRNAs, proteins, and metabolites associated with CCA presence. (G) Treeplot of KEGG pathways enriched across variables identified by WGCNA and DIABLO analysis. Pathways are grouped by similarity and ranked by adjusted significance and presence across all omic layers. (H) Heatmap summarizing the predominant contributors within each enriched pathway cluster. Only clusters supported by at least 2 terms are displayed. *Isoforms of proteins. Abbreviations: ALP, alkaline phosphatase; CCA, cholangiocarcinoma; DIABLO, Data Integration Analysis for Biomarker discovery using latent variable approaches in omics studies; IBD, inflammatory bowel disease; KEGG, Kyoto Encyclopedia of Genes and Genomes; ML, machine learning; PSC, primary sclerosing cholangitis; WGCNA, Weighted Gene Co-expression Network Analysis.

Network and ML-based data integration highlighted coordinated changes in 56 features, including extracellular matrix-related proteins, regulatory miRNAs, and detoxification-associated metabolites in PSC-CCA (Figure 5D-E). We identified several miRNAs with well-established roles in CCA pathogenesis, including members of the miR-15 and miR-29 families, as well as miR-24-3p, miR-101-3p, and miR-376a/c (Figure 5D and Supplemental S9, http://links.lww.com/HC9/C412). Some of these miRNAs are known regulators of extracellular matrix remodeling and epithelial–mesenchymal transition and have been functionally linked to TP53, KRAS, and FGFR2 signaling networks in CCA and other biliary cancers.^28^^,^^29^ For example, miR-29 suppresses collagen synthesis downstream of the TP53 and TGF-β pathways,^30^ whereas miR-15 targets anti-apoptotic and cell cycle genes modulated by KRAS activation.^31^ In addition, miR-24-3p and miR-101 further regulate MAPK-driven and FGFR2-driven proliferation and invasion programs,^29^^,^^32^ consistent with their enrichment in PSC-CCA plasma. At the metabolomic level, we identified a dense, co-regulated cluster of sulfated phenolics and methylxanthines (eg, 3-acetylphenol sulfate, 3-methoxycatechol sulfate, hippurate, caffeine, etc.) that was significantly reduced in PSC-CCA (Figure 5D and Supplemental S9, http://links.lww.com/HC9/C412). These metabolites are hepatic conjugation products, and their consistent downregulation suggests impaired phase II detoxification and xenobiotic clearance, reflecting the metabolic reprogramming characteristics of *IDH1/2*-mutant and *TP53*-deficient tumors.^33^ Finally, alterations in the protein level converged on inflammation and cytokine signaling (IL6), extracellular matrix (BGN, ITGA11, ITGB1), and microenvironment remodeling (CCL20, SPINK1, ANGPTL4) in patients with PSC who progressed to CCA. Several of these proteins (eg, IL6, ANGPTL4, and SPINK1) have been previously implicated in CCA,^34^^–^^36^ whereas others, such as BGN, ITGB1, and ITGA11, represent novel associations that highlight alterations in the extracellular matrix that contribute to tissue remodeling and malignant progression. Cross-layer correlation analysis revealed a coordinated variation of methylxanthines and sulfated phenolics, including caffeine, paraxanthine, trigonelline, and catechol sulfates, resulting in a loss of hepatic detoxification capacity (Figure 5F and Supplemental S6B, http://links.lww.com/HC9/C409). Conversely, coupled changes in membrane-trafficking proteins (SNAP23, SCAMP3, STX8), adaptor PAG1, sphingolipids (sphingosine, sphinganine), and regulatory miRNAs (let-7f-5p, miR-4753) suggest shifts in vesicle dynamics and pro-tumor signaling.^37^ Co-variation of miR-15a/15b family members with 3-methoxycatechol sulfate aligned with dysregulated cell-cycle and apoptotic control during malignant progression, consistent with the reported roles of miR-15a/15b in cell-cycle arrest and apoptosis.^38^ Finally, consistent with earlier observations of PSC severity, pathway analysis revealed that biological perturbations converge on infection-related and malignancy-related mechanisms (cluster 1, Figure 5G, and Supplement S7, http://links.lww.com/HC9/C410). Notably, although the overarching pathways were shared, the leading parameters (terms) differed, suggesting a potential mechanistic shift within the same biological pathways.

Overall, PSC-CCA was characterized by multi-omic alterations, including miRNA regulators of extracellular matrix remodeling, epithelial–mesenchymal transition, and cell cycle, reduced sulfated phenolics and methylxanthines, and proteins involved in growth factor signaling, highlighting coordinated shifts in inflammation, microenvironment, and metabolism with both known and novel CCA-associated features.

## DISCUSSION

To our knowledge, this is the first study to provide integrative profiling of circulating miRNA, proteomic, and metabolomic datasets in patients with PSC and PSC-associated CCA. Combined analysis revealed coordinated miRNA–protein–metabolite modules that biologically characterized the clinical phenotypes and may ultimately support the development of minimally invasive, multi-analyte biomarkers for early CCA detection and PSC monitoring.

A challenge inherent to PSC research is the rarity of biospecimens, especially for rare subgroups such as PSC without IBD, estimated at ~20% of PSC cases,^39^ and even more so for PSC-CCA. This restricts statistical power but highlights the critical need for multi-omic approaches to maximize information yield from limited, high-value cohorts. Although scalability remains challenging, the initial success in biomarker identification justifies further investment in expanded datasets for replication and validation.

Our main findings were distinct circulating signatures of PSC-CCA and advanced PSC. The dominant metabolic signal in PSC-CCA was a consistent depletion of sulfated phenolic compounds and methylxanthines (eg, caffeine, paraxanthine, 3-acetylphenol sulfate), suggesting impaired hepatic conjugation and xenobiotic metabolism, in accordance with previous studies.^40^ Together with miRNA regulators of extracellular matrix remodeling, epithelial–mesenchymal transition, and proliferative signaling, these findings align with the metabolic reprogramming commonly observed in *IDH1/2*- or *TP53*-altered cancers.^33^ miRNA dysregulation is well documented in CCA,^41^ reproducible circulating biomarkers for clinical use remain elusive.^42^ Proteomic and metabolomic studies of PSC and CCA have identified candidate markers, but replication across cohorts is limited.^43^^–^^46^ The present analysis extends this work by identifying an integrated multi-omic module in PSC patients with CCA.

Severe PSC is characterized by a distinct circulating molecular profile that reflects inflammatory, fibrotic, and metabolic remodeling. Although these pathways overlap with those implicated in cholangiocarcinogenesis, further validation is required to determine their potential as biomarkers of disease complications and cancer risk. Like the PSC-CCA signature, malignant transformation in PSC may arise through gradual amplification of inflammatory, fibrotic, and metabolic pathways that are already active in advanced disease rather than through abrupt oncogenic events. Several studies have collectively revealed that extracellular RNAs, immune-inflammatory proteins, and serum metabolite signatures characterize PSC and malignant transformation to CCA. Previously reported dysregulated circulating miRNAs in PSC are consistent with our findings. These include miR-26a, the miR-29 and let-7 families and miR-223-3p, miR-15b, and miR-126. In addition, proposed diagnostic biomarkers such as miR-24-3p and the miR-30 family, as well as therapeutic candidates such as miR-122 and miR-885 in CCA, have also been identified.^47^^–^^49^ Proteomic surveys of serum and extracellular vesicles have identified candidate biomarkers, concordant with our findings. These include ADGRE2, DCBLD2, and S100A4, which are involved in extracellular matrix remodeling, angiogenesis, and tumor invasion.^50^^,^^51^ Our data confirmed elevated levels of conjugated primary bile acids, notably glycocholate, taurocholate, glycochenodeoxycholate, and taurochenodeoxycholate, as well as disturbances in lipid and amino-acid metabolism, as reflected by increased levels of di-hydroxybutyrate, alanine, creatinine, and leucine.^52^

Beyond reproducing single-omic findings, our study connects molecular layers within an integrated framework, linking credible biomarkers and therapeutic targets across the miRNA, proteomic, and metabolic dimensions. This integrative approach not only consolidates previous discoveries but also enables the exploration of cross-omic interactions underlying PSC progression and CCA development. Ultimately, it offers a foundation for multi-analyte biomarker panels and system-level therapeutic strategies.

Another key asset of this study is the demonstration that ML-based integration can uncover subtle yet coordinated molecular differences exemplified here by the differences found between PSC with and without IBD. Unlike correlation-based network methods that rely on strong linear associations, ML approaches such as DIABLO combine dimensionality reduction with supervised feature selection, thereby detecting complex, multivariate patterns across omic layers. This enhances sensitivity to small-effect molecular variations, enabling robust molecular stratification, even in small, heterogeneous clinical datasets. Our findings highlight the complementary strengths of multi-omics and ML-driven integration in uncovering complex disease-defining molecular signatures. The resulting signatures capture both shared and trait-specific processes from immune activation and extracellular matrix remodeling to metabolic reprogramming, reflecting the continuum from inflammation to malignancy.

However, a key limitation of this study is the limited number of cholangiocarcinoma cases available for analysis (n=6), reflecting the rarity of CCA within this cohort. The small subgroup size restricts statistical power and limits the robustness and generalizability of subgroup-specific findings, particularly for analyses comparing IBD-associated or PSC-associated disease. Consequently, the identified molecular associations should be interpreted as exploratory and hypothesis-generating. Future studies in larger, independent cohorts will be necessary to validate these observations and determine their broader applicability.

Despite these limitations, the multi-omics framework provides insights into molecular alterations that may contribute to the pathogenesis of CCA in inflammatory hepatobiliary disease contexts. Several of the identified molecular features are involved in pathways related to inflammation, metabolic reprogramming, and tumor-associated signaling, processes that have been implicated in the progression from chronic biliary inflammation to malignant transformation. These observations support the concept that inflammatory microenvironments, such as those present in PSC and IBD, may shape tumor-associated molecular profiles. While further validation is required, the identified molecular signatures may ultimately contribute to improved mechanistic understanding and could inform the development of biomarkers or therapeutic targets in inflammation-associated CCA.

## CONCLUSION

This study highlights the potential of circulating multi-omic markers, including miRNAs, proteins, and metabolites, to provide an integrated molecular overview of PSC activity and the risk of complications. These results underscore the potential of circulating omics as a minimally invasive biomarker that can complement current strategies for cancer risk stratification, prognosis, and monitoring in PSC, with further validation in independent, larger cohorts.

## Supplementary Material
